# Supplementary material for: Physical understanding of the extreme global temperature jump in 2023
Source: Commun Earth Environ. 2026 Mar 19;7(1):406. doi: 10.1038/s43247-026-03382-6 (PMC13167450; doi:10.1038/s43247-026-03382-6)
Supplement: Supplementary file 2 — Supplementary Material [file 43247_2026_3382_MOESM2_ESM.pdf]

Supporting Information for

Physical understanding of the extreme global temperature jump in 2023

J. Mex[1,2], C. Cassou[1], A. Jézéquel[1,3], S. Bony[4], C. Deser[5]

[1]LMD/IPSL, École Normale Supérieure, PSL Université Paris, Paris, France

[2]Leipzig Institute for Meteorology, Leipzig University, Leipzig, Germany

[3]École Nationale des Ponts et Chaussées, Champs sur Marne, France

[4]LMD/IPSL, Sorbonne University, CNRS, Paris, France

[5]National Center for Atmospheric Research, Boulder, CO, USA.

Contents of this file

Figures S1 to S7

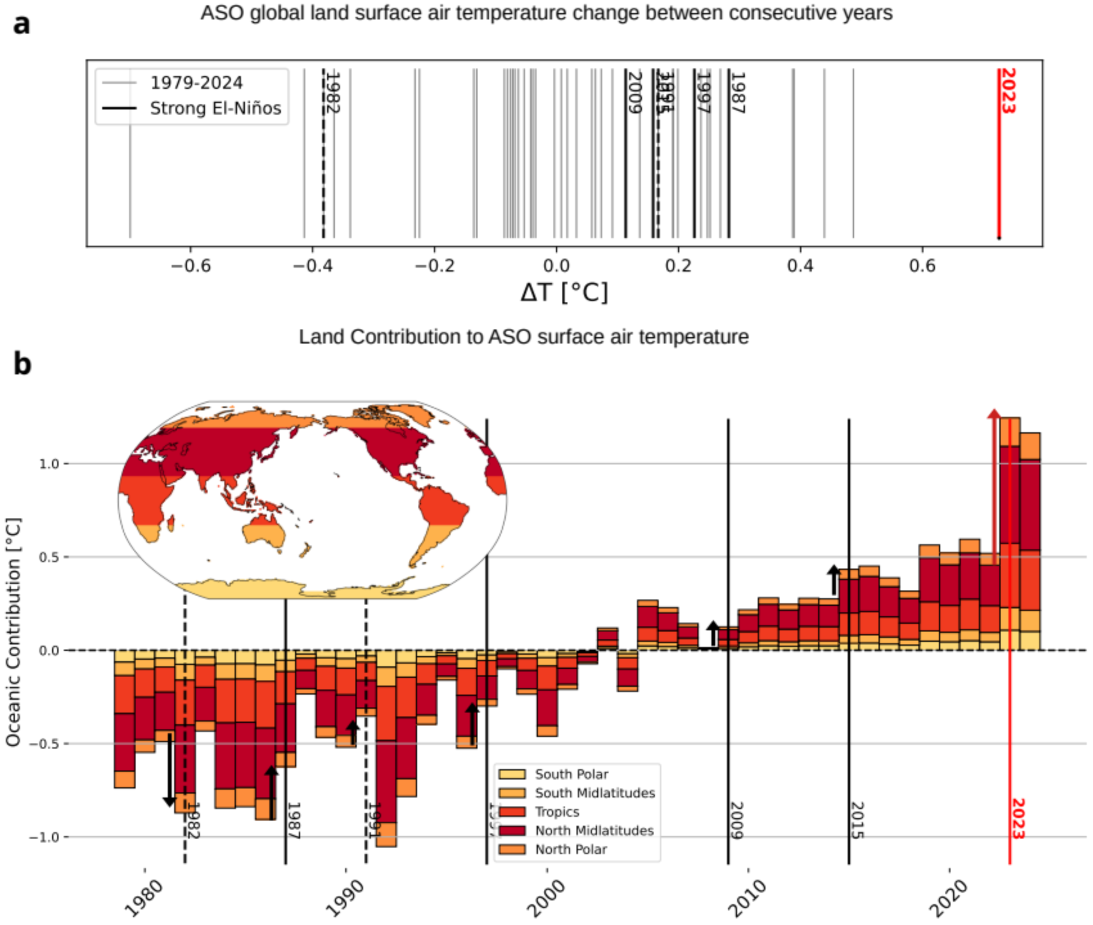

Figure S1. Observed record-breaking Global Land Surface Air Temperature (GLSAT)

a) As Figure 1a) for global marine surface air temperature but for global land. b) As Figure 1b) for contributions of land surface in area-weighted latitudinal bands.

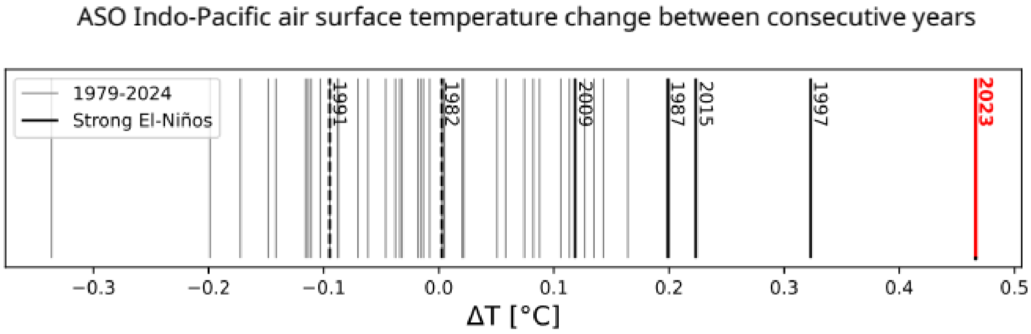

Figure S2. The year-to-year jump in Indo-Pacific marine Surface Air Temperature. As Figure 1a) but for the Indo-Pacific marine surface air temperature.

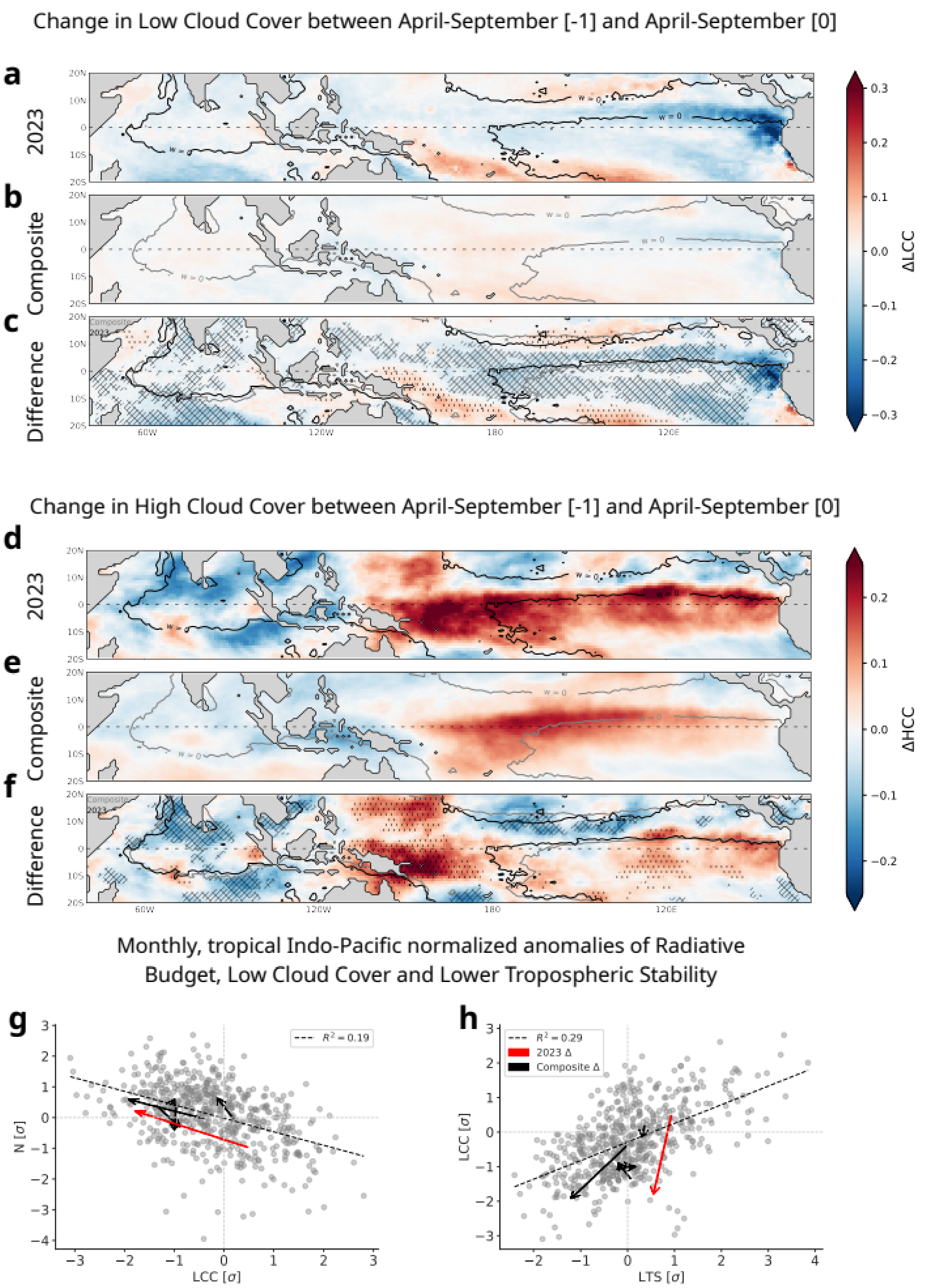
Change in Low Cloud Cover between April-September [-1] and April-September [0]
a
2023
b
Composite
c
Difference
ΔLCC
Change in High Cloud Cover between April-September [-1] and April-September [0]
d
2023
e
Composite
f
Difference
ΔHCC
60W
120W
180
120E
Monthly, tropical Indo-Pacific normalized anomalies of Radiative Budget, Low Cloud Cover and Lower Tropospheric Stability
g
$R^2 = 0.19$
N [σ]
LCC [σ]
h
$R^2 = 0.29$
2023 Δ
Composite Δ
LCC [σ]
LTS [σ]

Figure S3. Changes in cloud cover for the 2023 El Niño built-up. Changes in Cloud Cover prior to ASO, between AMJJAS [0] and AMJJAS [-1] for 2023 (a, d), the composite (b, e) and the difference between the two (c, f) for the low cloud cover (LCC, a-c) and high cloud cover (HCC, d-f) . Cross shading is used in regions where 2022-2023 is lower than all of the composite years, stippling in regions where it is higher. **g)** Scattered in light grey are monthly standardised anomalies of tropical (S20-N20), indo-pacific TOA radiative budget against tropical indo-pacific low cloud cover along with its linear regression line (dotted) (R^2 = 0.19) for the time period of 1979-2023. Arrows indicate the change between two consecutive years: the arrow points from the AMJJAS [-1] average to the AMJJAS [0] average for all the strong El Niños (black) in the composite and 2023 (red). **h)** as in **g)** but for tropical indo-pacific low cloud cover against tropical indo-pacific lower tropospheric stability (LTS) with their linear regression shown (R^2 = 0.29).

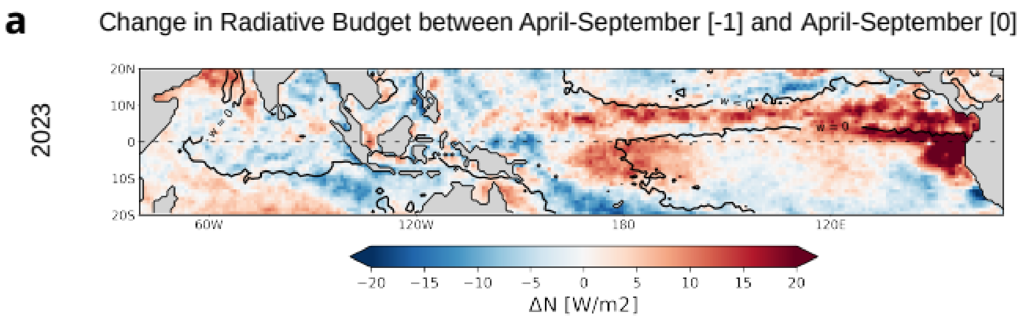

Figure S4. CERES top of atmosphere net radiation changes. As Figure 2 b) but with CERES-EBAF data.

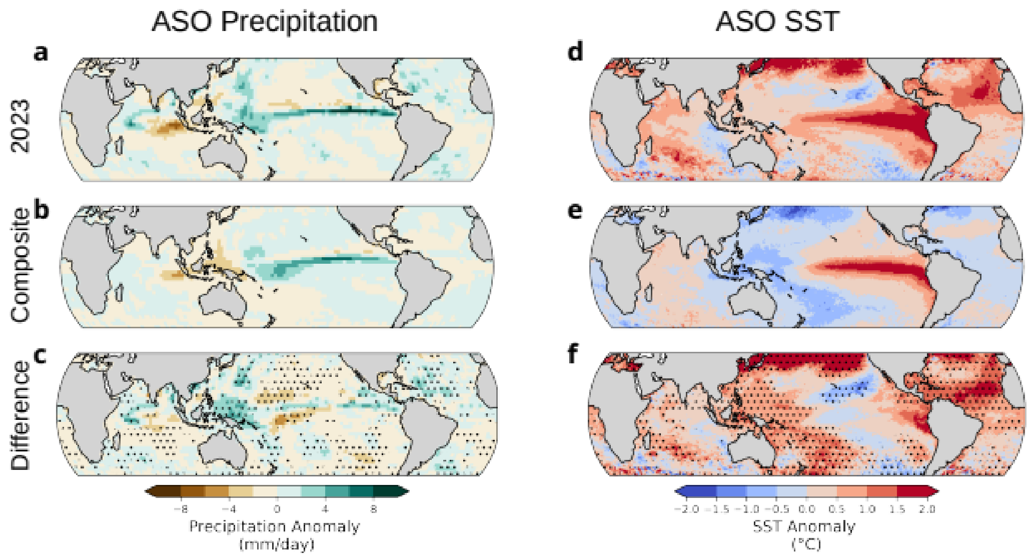

Figure S5. Specificity of the 2023 ENSO event in ASO. Precipitation (left) and SST (right) anomalies for 2023 (top row) with respect to the 1991-2020 climatology, for ENSO composite of strong El-Niño years (1987, 1991, 1982, 1997, 2009, 2015) (middle row) and the difference between the two (bottom row). Stippling indicates that the 2023 anomalies are the largest of any ENSO events used for compositing.

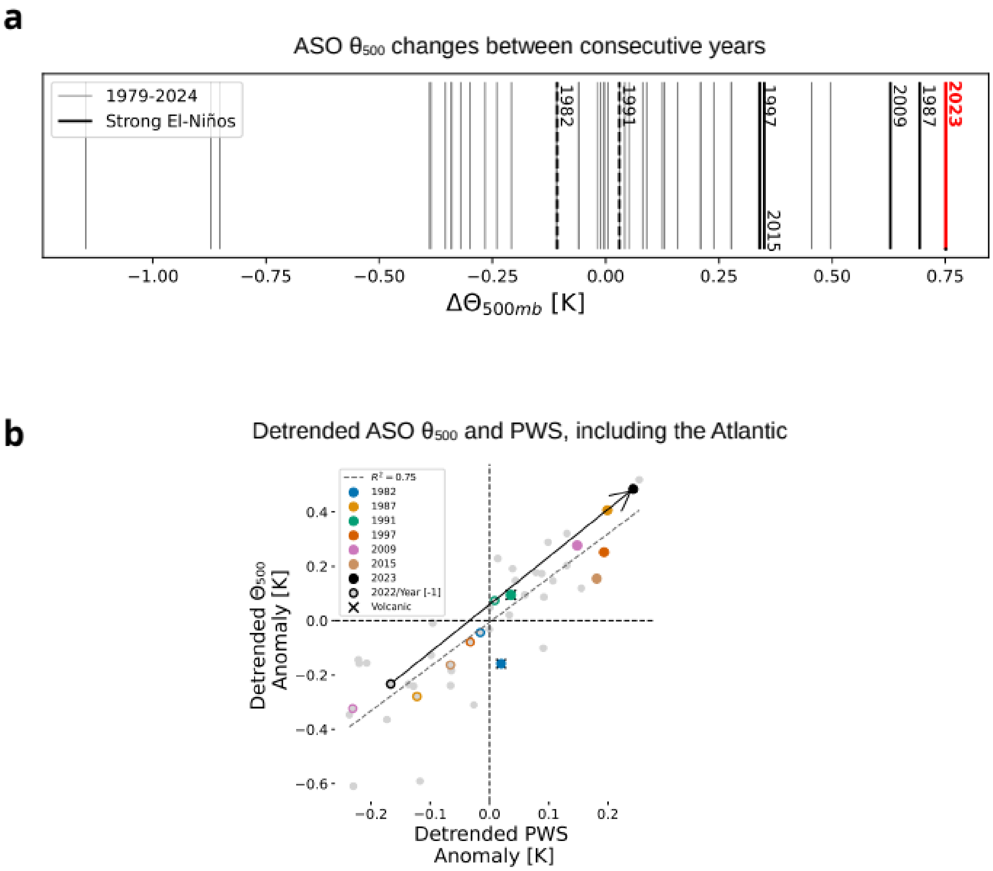

Figure S6. The record-breaking jump in tropospheric temperature and SST in deep convective regions. a) Change of tropical (S15-N25) average of ASO Θ_{500mb} between two consecutive years over 1979-2024 (grey), with strong El-Niños and 2023 highlighted in black and red, respectively. Dotted lines stand for El Niño years perturbed by strong volcanic eruptions. **b)** As Fig.4b b) including all tropical oceans for the calculation of PWS.

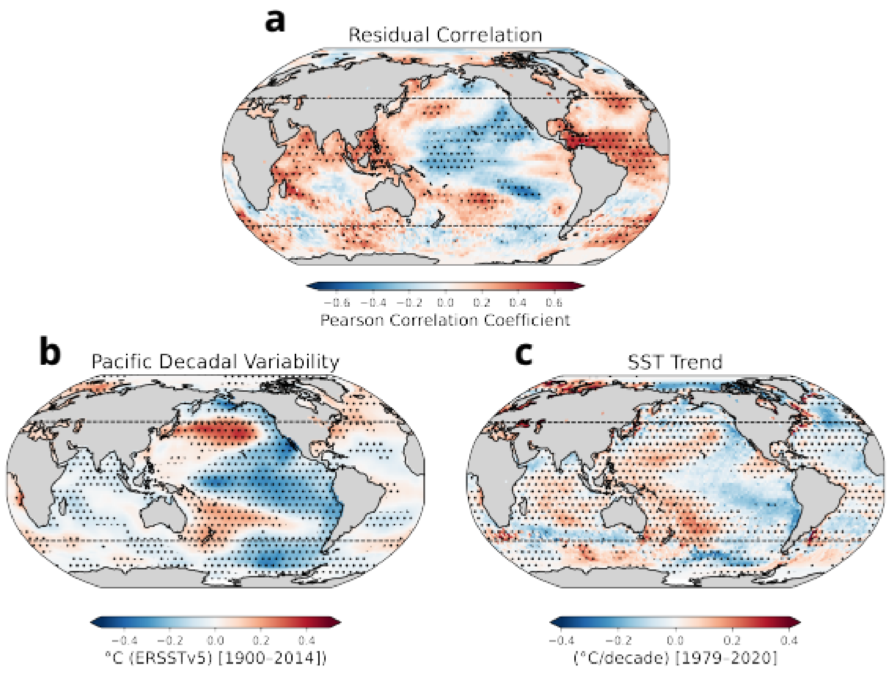

Figure S7. a) Oceanic regions of importance in the modulation of interannual tropospheric warming and the observed patterns of low-frequency SST changes. Correlation of residuals of ASO Θ_{500mb} onto SST as in Fig 4 c). **b)** Loading Pattern of Interdecadal Pacific Variability (negative phase) as defined by the IPCC AR6 (Cassou et al. 2021). **b)** ASO SST trend over the period 1979-2020. The pattern correlation coefficient is 0.42 between a) and b) and 0.17 and between a) and c). Dots indicate significance at the 10% level.
